# Interplay between Alba and Cren7 Regulates Chromatin Compaction in *Sulfolobus solfataricus*

**DOI:** 10.3390/biom12040481

**Published:** 2022-03-22

**Authors:** Marc Kenneth M. Cajili, Eloise I. Prieto

**Affiliations:** National Institute of Molecular Biology and Biotechnology, University of the Philippines, Diliman, Quezon City 1101, Philippines; mmcajili@up.edu.ph

**Keywords:** archaea, chromatin, chromosomal proteins

## Abstract

Chromatin compaction and regulation are essential processes for the normal function of all organisms, yet knowledge on how archaeal chromosomes are packed into higher-order structures inside the cell remains elusive. In this study, we investigated the role of archaeal architectural proteins Alba and Cren7 in chromatin folding and dynamics. Atomic force microscopy revealed that *Sulfolobus solfataricus* chromatin is composed of 28 nm fibers and 60 nm globular structures. In vitro reconstitution showed that Alba can mediate the formation of folded DNA structures in a concentration-dependent manner. Notably, it was demonstrated that Alba on its own can form higher-order structures with DNA. Meanwhile, Cren7 was observed to affect the formation of Alba-mediated higher-order chromatin structures. Overall, the results suggest an interplay between Alba and Cren7 in regulating chromatin compaction in archaea.

## 1. Introduction

The genomic material of all organisms must be packaged within the relatively smaller compartments in which they are confined, while at the same time, DNA accessibility must be maintained to accommodate processes necessary for normal cell function, such as replication and transcription. Biophysical processes such as DNA supercoiling and macromolecular crowding allow for the compaction of chromatin [1]. However, the regulation of genome accessibility is primarily facilitated by architectural proteins that aid in the compaction of the genome [1,2,3]. In eukaryotes, the architectural proteins primarily responsible for genome organization and dynamics are histones. These proteins form octamers which wrap around 150 bp of DNA into solenoidal coils [4,5]. In bacteria, several nucleoid-associated proteins (NAPs) regulate genome compaction. These NAPs can cause deformation of DNA through bending, wrapping, or bridging, forming stable loops in the genome that allow for greater compaction [6,7,8].

A similar process must exist in archaea, the third domain of life, given the necessity of genome compaction and regulation in the normal function of cells. Similarities between the information-processing systems in eukaryotes and archaea can lead one to infer that comparable genome organization mechanisms may exist between the two domains. This was confirmed with the discovery of histone homologues in *Methanothermus fervidus* [9] that were shown to resemble eukaryotic histones in terms of binding and compaction mechanisms [10,11]. Not all species of archaea were found to contain histones [11], however, and later studies showed the presence of NAPs in the phylum Crenarchaea that are comparable to their bacterial counterparts, suggesting that bacterial genome compaction mechanisms may be utilized by members of this phylum. Some of these NAPs include Sul7 [12], MC1 [13], Cren7 [14], and members of the Alba superfamily [3].

Most archaeal species were observed to have at least two DNA-binding architectural proteins [3,4]. A prevailing hypothesis, therefore, is that the interplay of more than one type of architectural protein, whether synergistic or antagonistic, is needed to mediate chromatin organization in archaea. Among the different architectural proteins, members of the Alba superfamily are the most prevalent among all species of archaea [3,4]. Alba is a 10 kDa protein that occurs as a homodimer in solution. It can bind to DNA without any sequence specificity, and it can mediate the formation of DNA bridges [15,16]. It was also observed in vitro that the binding affinity of Alba can be modulated through acetylation [17]. The extensive conservation of this protein in most species of archaea, its high level of expression in cells proximal to the nucleoid region [18], and its DNA-binding ability suggest that Alba may play an important role in archaeal chromatin organization.

Another protein of interest is Cren7, a relatively small 7 kDa protein that is widely distributed among members of the phylum Crenarchaeota [4]. It exists as a monomer in nature and, like Alba, is capable of non-specific binding to DNA [14]. These properties suggest an important role for Cren7 in the genome compaction of crenarchaea. Determining the mechanisms of the interaction and interplay between these two architectural proteins and DNA may provide better understanding of the chromatin compaction mechanism and regulation in the archaeal domain.

In this study, the roles of the architectural proteins Alba, which is conserved in most species of archaea, and Cren7, which is present in non-histone containing crenarchaea, in the compaction, organization, and regulation of the archaeal genome were characterized. Recombinant *Sulfolobus solfataricus* Alba and Cren7 heterologously expressed in *Escherichia coli* were used to reconstitute chromatin in vitro, and the resulting nucleoprotein structures were visualized using atomic force microscopy (AFM). Short linear 3 kb DNA was employed to determine local deformations caused by the proteins, while a longer 48.5 kb bacteriophage λ DNA was used to observe potential long-range interactions that may play a role in compaction. On-substrate lysis and AFM imaging was carried out to determine the structural details of the native *S. solfataricus* chromatin in comparison with the in vitro reconstituted samples. Lastly, potential changes in gene expression patterns of the architectural proteins and their regulators were characterized though quantitative PCR. By utilizing nanoscale analysis of in vitro reconstituted and native chromatin, combined with observed changes in the gene expression of proteins that facilitate post-translational modification, this study provides a possible mechanism for how *S. solfataricus* chromatin architectural proteins compact and remodel archaeal DNA, giving insight into the process of genome organization and dynamics in archaea.

## 2. Materials and Methods

### 2.1. Sulfolobus Solfataricus Strain and Growth Conditions

*Sulfolobus solfataricus* DSM 1616 (RIKEN Bioresource Research Center, Tsukuba, Japan) was grown in aerobic conditions at 80 °C in a medium containing modified Allen’s basal salt solution (3.1 g KH_2_PO_4_, 2.5 g (NH_4_)_2_SO_4_, 0.2 g MgSO_4_·7H_2_O, 0.25 g CaCl_2_·2H_2_O in 1 L H_2_O) [19,20] supplemented with 0.1% yeast extract and 0.1% casamino acids and adjusted to pH 4.0 using H_2_SO_4_.

### 2.2. Micrococcal Nuclease Digestion

*S. solfataricus* cells in exponential and stationary phase were harvested, resuspended in MNase digestion buffer (50 mM Tris-Cl, pH 7.5, 5 mM CaCl_2_, 0.5% Triton X), and incubated at 37 °C for 5 min. Each sample was divided into 4 tubes, and increasing amounts (0, 0.2, 0.5, and 1 unit) of micrococcal nuclease (Sigma Aldrich, St. Louis, MI, USA) were added to each tube, along with 200 μg/mL RNase A (Invitrogen, Waltham, MA, USA), followed by incubation at 37 °C for 15 min. The reaction was stopped with the addition of freshly prepared stop solution (2% SDS, 100 mM EDTA) and then incubated with 20 mg/mL Proteinase K (Invitrogen) at 58 °C for 1 h. DNA from each tube was isolated by phenol-chloroform extraction and subjected to electrophoresis through a 2.5% agarose gel at 100 V for 30 min. The gel was subsequently stained using 3X Gel-Red (Biotium, Fremont, CA, USA) and imaged.

### 2.3. On-Substrate Lysis

On-substrate lysis of cells was performed based on a previous study by Kim et al. [21] with modifications. *S. solfataricus* culture were harvested, washed, and deposited on a clean coverslip for 10 min in room temperature and dried using nitrogen gas. Cells were lysed through the addition of lysis buffer (10 mM Tris-Cl, pH 7.5, 10 mM EDTA, 0.5% Triton-X), followed by washing with sterile distilled/deionized water and drying with nitrogen gas. Samples were then imaged through atomic force microscopy using the XE-BIO system (Park Systems, Suwon, South Korea).

### 2.4. Expression and Purification of Recombinant Architectural Proteins

*S. solfataricus* Cren7 gene was amplified from the genomic DNA using the following primers with 5′ NdeI and 3′ XhoI restriction endonuclease site overhangs (for Cren7 forward: 5′-CAGAGGCATATGATGAGTTCGGGTAAAAAACCAGTA-3′; for Cren7 reverse: 5′-TCGACCCTCGAGTTATATTGGATAATCATCTGGTAG-3′). The amplicons were then inserted into an NdeI- and XhoI-(New England Biolabs, Ipswich, MA, USA) digested pET-30a expression vector (Novagen/Merck Biosciences, Darmstadt, Germany). The resulting construct, together with *S. solfataricus* Alba1 and Alba2 constructs obtained from Dr. Malcolm White (University of St. Andrews), were then transformed into chemically competent *Escherichia coli* BL21(DE3) cells (Novagen/Merck Biosciences, Darmstadt, Germany). Expression was induced at OD_600_ = 0.6 through the addition of 1 mM IPTG and subsequent incubation at 37 °C for 3 h.

The cells were harvested and subsequently resuspended in lysis buffer (50 mM Tris-Cl, pH 7.5, 100 mM NaCl, 1 mM EDTA, 1 mM DTT, 1 mM PMSF) followed by sonication to extract the recombinant proteins. The extracted proteins were then purified through ion-exchange chromatography using a 5 mL HiTrap SPHP cationic column (GE Healthcare/Amersham Biosciences, Chicago, USA). A gradient of 0 to 1 M NaCl in Buffer A (50 mm Tris-Cl pH 7.5, 1 mM EDTA, 1 mM DTT, 1 mM PMSF) was applied. The eluted proteins were pooled and further purified through size exclusion chromatography using a TSKgel G3000SWxl column (Tosoh Biosciences, Tokyo, Japan), with 150 mM NaCl in Buffer A serving as a mobile phase.

### 2.5. Formation of Alba Heterodimers

Alba1 and Alba2 have been previously determined to form obligate heterodimers at concentrations exceeding the respective proteins’ dissociation constants (<10 nM) [15]. Equimolar amounts (~100 µM) of Alba1 and Alba2 were added together to form the Alba heterodimer. To ensure successful heterodimer formation, the samples were run in size exclusion chromatography using a TSKgel G3000SWxl column (Tosoh Biosciences) with 150 mM NaCl in Buffer A as mobile phase.

### 2.6. In Vitro Chromatin Reconstitution

To determine the effect of the architectural proteins on DNA structure, reconstitution with a 3 kb DNA was performed. pGEM-T Easy vector (Promega, Madison, WI, USA) was digested overnight with EcoRI (Invitrogen) at 37 °C and purified using High Pure PCR Product Purification Kit (Roche, Basel, Switzerland). Recombinant proteins were then added with the linearized DNA at various protein-to-DNA weight ratios in AFM Buffer A (100 mM NaCl, 10 mM HEPES, pH 8.0). The set-ups were incubated for 10 min at 60 °C and subsequently fixed for 10 min at room temperature with fixation buffer (0.3% glutaraldehyde in AFM buffer A). The samples were then deposited onto freshly cleaved mica pre-treated with 100 mM spermidine for 10 min, rinsed with 2 mL Ultrapure™ water (Invitrogenand dried using nitrogen gas.

To characterize potential long-range interactions between chromatin proteins, reconstitution was performed following the protocol of Maurer et al. [22]. Briefly, 48.5 kb bacteriophage λ DNA (Invitrogen) was first added to AFM Buffer B (50 mM KCl, 20 mM HEPES pH 8.0, 2 mM NiCl_2_, 0.005% Tween-20). Protein samples were added accordingly to obtain the desired protein-to-DNA weight ratios. The samples were incubated for 10 min at 60 °C, fixed for 10 min at room temperature with fixation buffer (0.3% glutaraldehyde in AFM buffer B), deposited onto freshly cleaved mica disks for another 10 min, rinsed with 2 mL Ultrapure™ water (Invitrogen), and dried with nitrogen gas.

### 2.7. Atomic Force Microscopy

Imaging was carried out using a Park Systems XE-BIO Atomic Force Microscope in intermittent mode using a PointProbe^®^ Plus Non-Contact/Tapping Mode–High Resonance Frequency probe (Nanoworld, AG/Park Systems, Suwon, South Korea). The samples were imaged at a 512 × 512 square pixel resolution. All images were analyzed and processed using WSxM Develop 8.1 [23]. The images were flattened, and contrast was adjusted accordingly to observe different features, such as height, width, contour length, and end-to-end distance for the in vitro reconstitution experiments. Contour length pertains to the measurement along the stretch of DNA, while end-to-end distance pertains to the measurement of the shortest distance between the ends of DNA. Since the tip can affect the apparent measurement of width in the images produced, measurement of the width at half of the height of the structures was performed to increase accuracy [21].

### 2.8. RNA Extraction and Quantitative PCR

Total RNA of cells harvested at exponential and stationary phases were extracted using Quick-RNA Fungal/Bacterial Kit (Zymo Research, Irvine, CA, USA) and treated with DNAse I (Sigma Aldrich) to remove any DNA contaminants. One microgram of RNA was subjected to reverse transcription using High-Capacity cDNA Reverse Transcription Kit (Applied Biosystems, Waltham, MA, USA). Quantitative PCR was then performed with PowerUP™ SYBR Green Master Mix (Applied Biosystems) and the primers listed in Table 1 using an Applied Biosystems 7500 Fast PCR system for 40 cycles (95 °C for 15 s, 60 °C for 1 min, 72 °C for 15 s). Relative expression levels were determined by making a standard curve using pooled cDNA. Expression levels were normalized to *S. solfataricus* GlyA gene.

## 3. Results

### 3.1. On-Substrate Lysis Reveals Fundamental Structure of the Nucleoid

**Figure 1:**
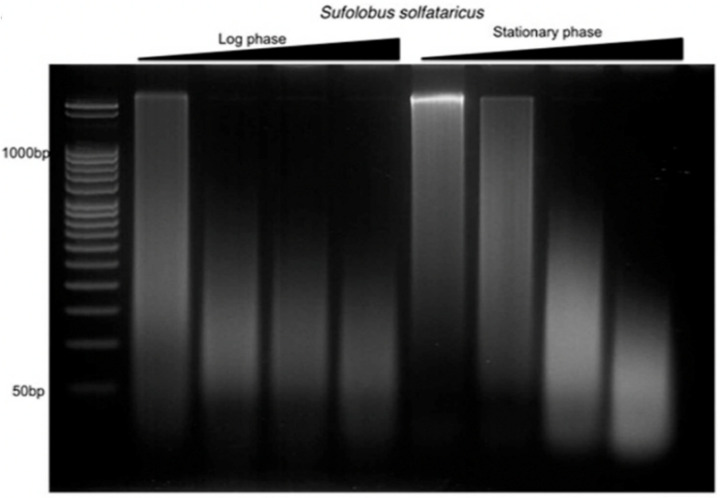
Micrococcal nuclease digestion of *S. solfataricus* nucleoid from logarithmic and stationary growth phases. The absence of distinct, regularly repeating structures was observed in all reactions.

To characterize the structural features of the archaeal nucleoid, *S.solfataricus* chromatin obtained from both exponential and stationary phase cultures was digested with micrococcal nuclease (MNase). Digestion is partly blocked by chromatin proteins giving rise to protected DNA fragments. These fragments can be analyzed to determine the smallest DNA length or minimal unit of the archaeal chromosome [24]. In addition, multiples of the minimal chromosome unit can reflect regularly spaced chromatin proteins along the DNA [25]. The lack of a distinct ladder pattern was noted for *S.solfataricus* (Figure 1), in contrast to what was previously observed in histone-encoding archaea [26,27]. This shows the absence of defined structural units that can protect against MNase digestion, which is consistent with previous observations on other species of crenarchaeotes [27].

**Figure 2:**
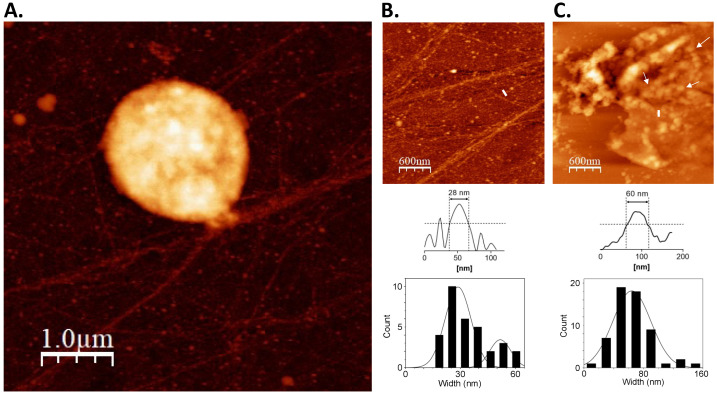
Nucleoid structure of *S. solfataricus*. (**A**) Partial lysis reveals the nucleoid structure from exponential phase *S. solfataricus* cells. Closer observation shows the presence of (**B**) 28 nm fiber structures and (**C**) 60 nm globule-like structures. The white arrows point to loops formed by the globule structures. The white line represents the line profile beside each image, and the broken line indicates the half-maximum height. A Gaussian curve was fitted with each histogram generated from width measurements (*n* = 30).

The MNase digestion profile was then corroborated through on-substrate lysis. This technique was performed to elucidate the in vivo chromatin arrangement of *S. solfataricus*. Knowing that the nucleoid is fragile, and steps during isolation and purification can alter any structural features present [21], the cells were mildly lysed on top of glass substrate to facilitate the release of cellular components, particularly the nucleoid. This was followed by AFM imaging without any purification, fixation, or staining steps, enabling the observation of the topographical details of the chromatin structure of *S. solfataricus* in a near-native state (Figure 2). Minute details of the chromatin structure were seen upon closer inspection of the released cellular components. These structures were posited to be smaller units of the *S. solfataricus* nucleoid. Globules and fiber-like structures were observed from the exponential phase nucleoid, confirming that the chromatin is loosened by partial lysis treatment (Figure 2). Focusing on the structures revealed the nucleoid to be composed of 60 nm globular particles (Figure 2C). The presence of 28 nm fibrous structures was also noted (Figure 2B). The globular particles in partially lysed cells also formed loop structures (Figure 2C). These suggest that the 28 nm fiber may form the basic structural unit of *S. solfataricus* chromatin. In turn, interactions between 28 nm fibers may give rise to higher-order structures such as the observed globule-like structures from which the fibers originate.

### 3.2. Alba Mediates the Formation of Regularly Structured Complexes while Cren7 Forms Loose Nucleoprotein Structures with Long DNA

**Figure 3:**
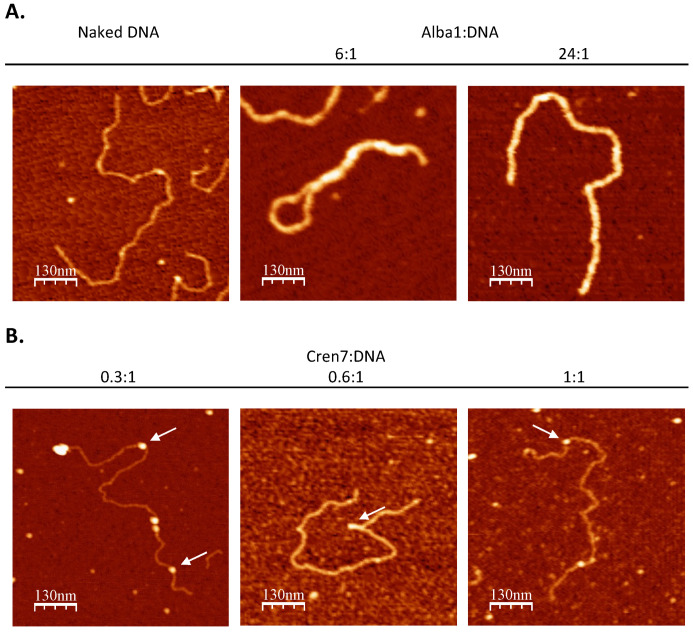
Structural effect of chromatin architectural proteins on DNA. AFM images of nucleoprotein complexes formed by 3 kb linear DNA with architectural proteins (**A**) Alba1 and (**B**) Cren7 at increasing protein-to-DNA weight ratios as indicated. Deformation induced by Cren7 is indicated by the white arrows.

In vitro chromatin reconstitution using architectural proteins Alba and Cren7 with a linearized 3 kb pGEM-T Easy vector showed the effect of each protein on the DNA structure. Alba induced a concentration-dependent change in DNA structure (Figure 3A). This is consistent with previous findings that demonstrated low amounts of Alba creating bridged DNA duplexes, while saturating amounts result in a more rigid structure [27,28,29]. Meanwhile, reconstitution with Cren7 induced bending along the DNA duplex (Figure 3B), congruent with previous studies [30,31]. Contour length and end-to-end distance measurements confirmed the extent of compaction mediated by each protein (Table 2). Contour length and end-to-end distance were at a minimum in the 6:1 Alba1-DNA set-up. On the other hand, these values were similar to naked DNA in the 24:1 Alba1-DNA and Cren7-DNA set-ups, demonstrating the absence of compaction in the nucleoproteins formed.

**Figure 4:**
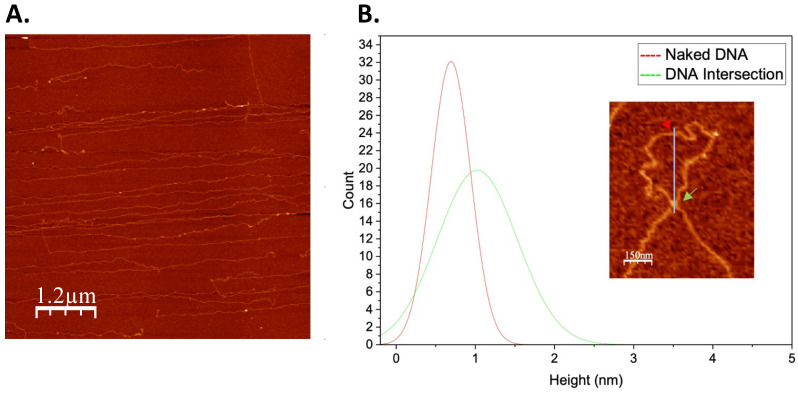
Height measurement of naked DNA and DNA intersections. (**A**) Representative AFM image of naked 48.5 kb bacteriophage λ DNA. Note the lack of any distinct structures. (**B**) Histogram (*n* = 100) of height of naked DNA and of DNA intersections. The height of naked DNA was measured to be 0.69 ± 0.25 nm, and the height of DNA intersections was determined to be 1.02 ± 0.50 nm. This difference in height allows structures to be distinguished in AFM images.

While in vitro reconstitution with short DNA is sufficient to show how architectural proteins affect local DNA structure, long-range interactions occurring within a compact nucleoid may be overlooked with this method. Therefore, to determine the role of Alba and Cren7 on chromatin organization, in vitro chromatin reconstitution was carried out with longer 48.5 kb bacteriophage λ DNA. Naked DNA was imaged along with each set-up to avoid mistaking artifacts due to sample preparation as nucleoprotein features (Figure 4A). Height analysis was also carried out to distinguish DNA overlaps and intersections from true nucleoprotein structures (Figure 4B).

**Figure 5:**
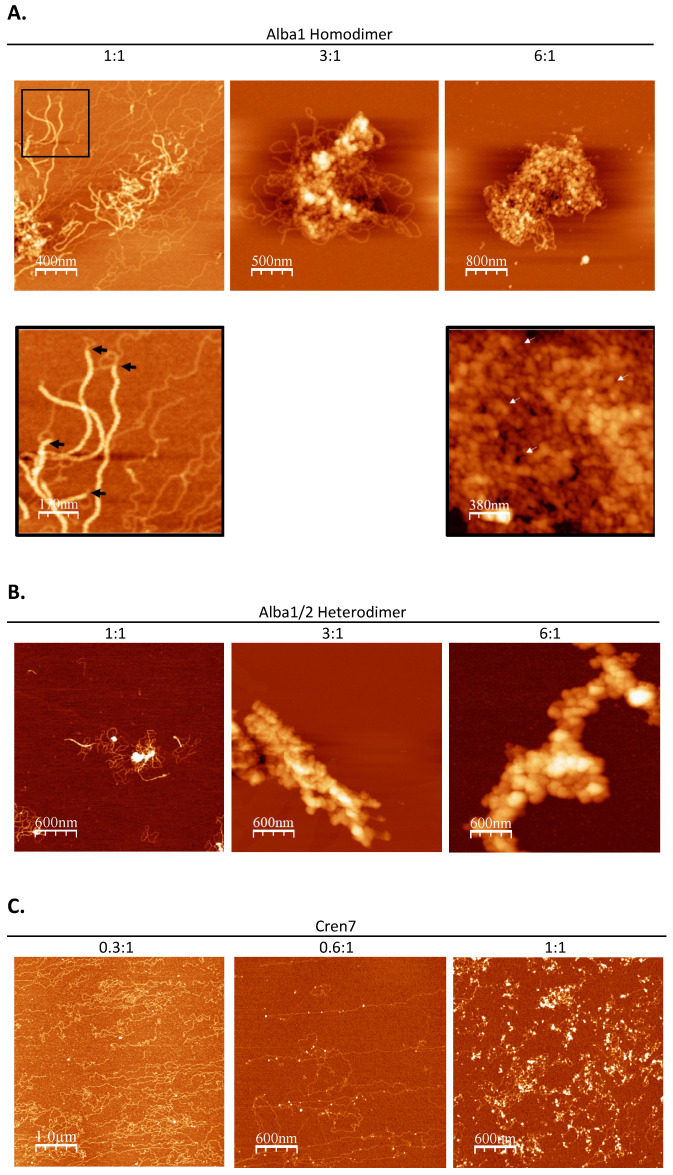
AFM images of complexes formed by a 48.5 kb bacteriophage λ DNA with architectural proteins (**A**) Alba1 homodimer, (**B**) Alba1/2 heterodimer, and (**C**) Cren7 at increasing protein-to-DNA weight ratio, as indicated. The black box presents a close-up image of filaments formed by bridged DNA duplexes (black arrow) and of loop structures formed by beads (white arrow).

Naked DNA was observed to be devoid of any distinct structural features (Figure 4A). Linear stretches of DNA with varying degrees of tangling were imaged. The addition of Alba1 homodimer at a weight ratio of 1:1 resulted in thick fiber structures of 21.52 ± 4.41 nm width and 2.99 ± 0.67 nm height (Figure 5A). Closer observation revealed that the fibers were composed of bridged DNA duplexes. Longer stretches of 20 nm nucleoprotein filaments were formed when the Alba:DNA weight ratio was increased to 3:1, indicating more extensive DNA bridging. In addition, parts of the nucleoprotein complexes adopted globular structures with a diameter of 43.23 ± 12.08 nm (Figure 5A). An overall more compact nucleoprotein complex composed predominantly of globular structures of similar diameter was generated at 6:1 Alba:DNA weight ratio. The globular particles were observed to form looped structures (Figure 5A). Together, these suggest the potential of Alba1 homodimer to compact DNA into complex higher-ordered structures.

*S. solfataricus* also encodes Alba2, a homologue of Alba that preferentially forms heterodimers with Alba1 [15]. The effect of Alba1/2 heterodimers on DNA compaction was also determined. In vitro reconstitution yielded nucleoprotein structures comparable to those obtained with the Alba1 homodimer (Figure 5B). Twenty-nanometer filaments composed of bridged DNA duplexes were formed at a 1:1 protein:DNA weight ratio. However, these structures were absent when the protein:DNA weight ratio was increased to 3:1 and 6:1. The nucleoprotein complexes formed at these ratios were mostly composed of compact and larger globular structures with a diameter of around 100 nm. These 100 nm beads were also seen to form looped structures similar to what was previously observed in the 6:1 Alba1:DNA set-up (Figure 5A). The formation of more compact and ordered nucleoprotein structures at lower protein concentrations indicates the ability of Alba2 to modulate the extent of Alba1-mediated compaction, possibly due to the difference in DNA-binding behavior of the two Alba proteins [29].

In vitro reconstitution using 48.5 kb bacteriophage λ DNA was also performed with Cren7 protein to determine its contribution to archaeal chromatin structure. DNA bends were visualized in the presence of Cren7 (Figure 5C), consistent with those observed in the short DNA set-up (Figure 3B) and other reconstitution studies [3,14]. However, increasing the amount of Cren7 resulted in the formation of loose nucleoprotein structures unlike the structures formed in the presence of Alba. This demonstrates that Cren7 can induce deformations in the DNA duplex but not to mediate long-range interactions to form highly compact and ordered structures on its own.

### 3.3. Interplay between Architectural Proteins Can Alter Chromatin Structures In Vitro

**Figure 6:**
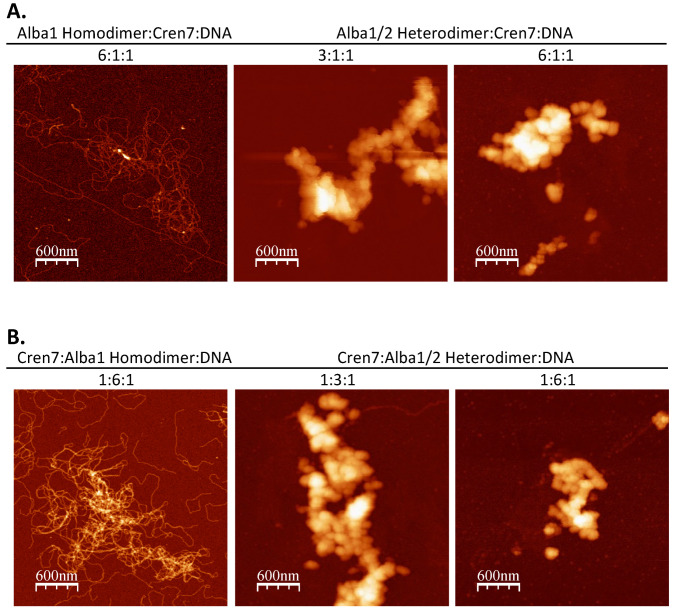
AFM images of λ DNA incubated with (**A**) Cren7 following the addition of Alba1 homodimer and Alba1/2 heterodimer and (**B**) Alba1 homodimer and Alba1/2 heterodimer following the addition of Cren7 at the indicated protein:DNA weight ratio (*w*/*w*/*w*). An overall looser nucleoprotein structure was observed upon the addition of Cren7 and Alba1 homodimer to the set-up regardless of order, while no significant structural changes were observed in set-ups containing Cren7 and Alba1/2 heterodimer.

Pairwise combinations of the different architectural proteins were utilized for in vitro chromatin reconstitution to investigate possible interplay between them. Addition of both Alba1 homodimer and Cren7 resulted in the formation of structures distinct from those found in the individual Alba1 homodimer-only and Cren7-only setups (Figure 6A, left panel). Overall, a looser nucleoprotein structure was observed in the presence of Cren7 regardless of the amount of Alba added. While 20 nm filaments similar to those found in Alba1 homodimer-only set-ups were observed, there was a notable decrease in the amount of compact globular structures as the amount of Alba1 homodimer increased (3:1 and 6:1 set-ups). This shows that the presence of Cren7 may potentially affect the formation of Alba-mediated higher-order chromatin structures. Meanwhile, when Cren7 and Alba1/2 heterodimer were utilized for chromatin reconstitution, highly compact nucleoprotein structures were formed similar to those seen in the Alba1/2 heterodimer-only set-up (Figure 6A, middle and right panels). This distinction with the homodimer-Cren7 set-up illustrates that Alba2 likely reinforces higher-order chromatin structures. Structural differences between Alba1 homodimer and Alba1/2 heterodimer may have corresponding effects on Cren7 binding, thereby preventing the formation of more open chromatin complexes with Cren7. Similar structures were observed regardless of the sequence in which the proteins were added during chromatin reconstitution (Figure 6B).

**Figure 7:**
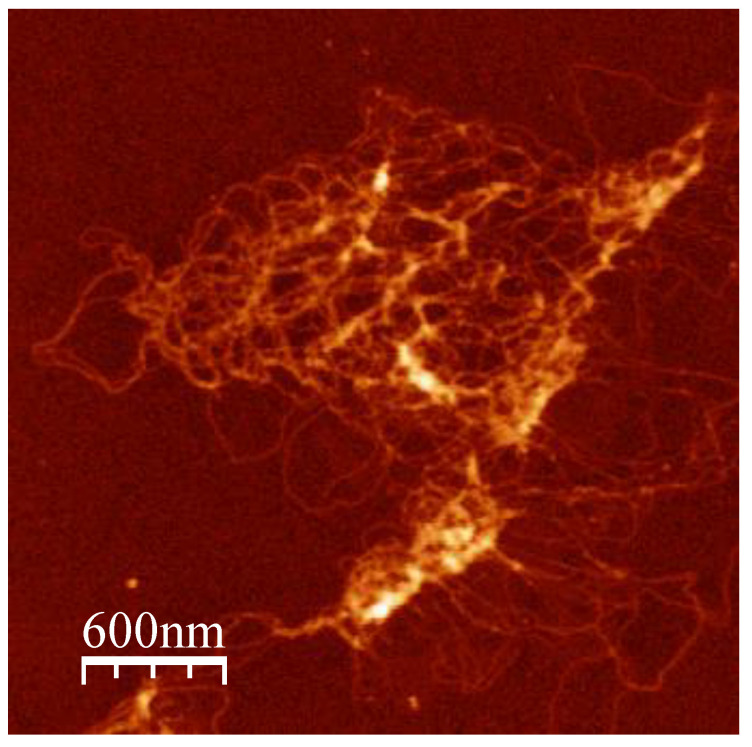
AFM image of λ DNA incubated with 6:3:1:1 (*w*/*w*/*w*/*w*) Alba1 homodimer:Alba1/2 heterodimer:Cren7:DNA. While an overall loose nucleoprotein structure was observed, there is a marked increase of compaction along some areas of the DNA.

To further explore the interplay between the proteins, chromatin containing Alba1 homodimer, Alba1/2 heterodimer, and Cren7 were prepared with λ DNA (Figure 7). Notably, the combination of the three proteins resulted in the formation of compact structures, albeit in a limited manner with respect to Alba-only set-ups. This supports the different roles of Alba and Cren7 in combination when mediating chromatin dynamics. Alba reinforces chromatin compaction, while the presence of Cren7 disrupts the organized structures formed by Alba protein, facilitating the formation of a more open chromatin structure.

### 3.4. Expression Level of Architectural Protein Regulators Changes with Growth Phase

**Figure 8:**
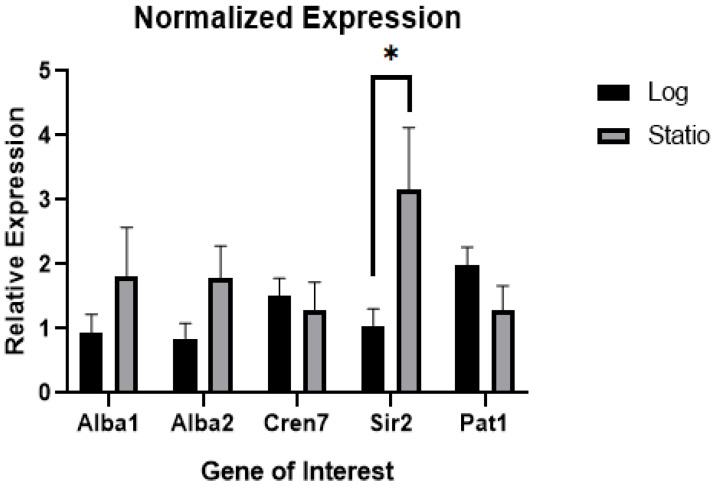
Expression levels of architectural protein genes for Alba1, Alba2, and Cren7, and enzymes mediating post-translational modifications (Sir2 and Pat1). A significant change in expression between exponential and stationary phase was observed in *Sir2*. Data corresponds to mean ± SD. Statistical significance between set-ups was determined through Welch’s *t*-test, * *p* < 0.05.

The results from the in vitro reconstitution experiments signified that the formation of higher-order structures, and consequently compaction, can be regulated by varying the amount of architectural proteins present. Additionally, the binding of these proteins to DNA may also be influenced by changes in binding affinity brought about by post-translational modifications (PTMs). For example, the binding activity of Alba proteins was determined to be dependent on their acetylation [16]. A higher degree of acetylation was shown to decrease the binding affinity of Alba with double-stranded DNA. Previous studies have also shown that acetylation in Alba is mediated through the deacetylase Sir2 [17] and protein acetyltransferase Pat [32]. To characterize the mechanism of chromatin dynamics in archaea, the gene expression of architectural proteins and the proteins involved in post-translational modifications were measured across different *S. solfataricus* growth phases, as previous studies have shown that nucleoid compaction varies between growth phases [33]. A significant increase in expression of Sir2 and a decrease in the expression pattern of Pat genes (Figure 8) were observed as cells transition from exponential to stationary phase. It is also noted that both Alba and Cren7 genes have similar expression levels between growth phases.

## 4. Discussion

In this study, the details and mechanism of chromatin organization and dynamics were analyzed in *Sulfolobus solfataricus*. We provided evidence that *S. solfataricus* chromatin consists of higher-order structures in the form of 60 nm globular beads. Moreover, in vitro reconstitution experiments have shown highly compact structures formed with Alba protein alone. Alba-mediated compaction was shown to be regulated by the DNA bender Cren7.

### 4.1. Architectural Landscape of the S. solfataricus Chromatin

Genome organization and the process of its regulation has been well-characterized in bacteria and eukaryotes. In eukaryotes, a hierarchical assembly is present, with the linker histone H1 facilitating the formation of higher-order 30 nm fibers from the 11 nm “beads-on-a-string” structure [34]. Likewise in bacteria, a similar hierarchical organization has been observed, such as in *E. coli*, whose nucleoid is composed of fundamental structures in the form of 40 nm and 80 nm fibers [21]. Results from on-substrate lysis showed a comparable stepwise assembly in *S. solfataricus*, with the presence of 28 nm fibers and 60 nm beads. This type of organization is similar to that observed using a similar approach by Maruyama and colleagues [27]. Although the structures present in *S. solfataricus* are apparently larger compared to the 10 nm fibers and 30–40 nm beads observed in on-substrate lysis experiments on *Thermoplasma acidophilum* and *Pyrobaculum calidifontis*, the interval between the size of the structures is similar.

*S. solfataricus* is deficient in histones and, similar to other non-histone-containing archaea [35,36], lacks a regular repeating unit within its chromatin. This suggests that, like bacteria, the hierarchical order observed in the chromatin may be facilitated solely through NAPs. NAPs are known to serve a role in chromatin compaction both in bacteria [37] and archaea [4], primarily by inducing bends and bridging duplexes. However, how exactly these proteins function in hierarchical assembly is not yet determined. From the in vitro reconstitution experiments, Alba seems to mediate the formation of a distinct higher-order structure.

### 4.2. Alba-Mediated Chromatin Structures

The chromatin protein Alba is highly conserved across the archaeal domain [3,16]. Structural analysis of Alba-reconstituted chromatin in this study confirmed the concentration-dependent manner by which Alba compacts DNA [15,28,29]. Surprisingly, Alba on its own was able to form higher-order structures with long DNA, comparable to those observed in native chromatin of various archaeal species [26,38,39], including *S. solfataricus* (Figure 2C and Figure 5A,B). These highly compact structures were absent in the short 3 kb DNA, implying that long-range interactions are involved in Alba-mediated higher-order chromatin folding. It is notable that this ability has been reported in vitro for neither eukaryotic histones [40] nor bacterial NAPs alone [22]. Combined with the knowledge that Alba is conserved across most species of archaea [41], this suggests that Alba is likely involved in a common chromatin compaction mechanism among non-histone containing archaea. It must be noted however that these higher order structures may be distinct from the larger chromosomal interaction domains (CIDs) previously observed in *Sulfolobus* in vivo [42]. Instead, these structures may serve another layer of organization connecting the structures formed by NAPs with larger domains, and it will be interesting to find how the Alba-mediated structures fit into the hierarchy.

Many archaeal species are known to encode multiple homologues of Alba [41]. In *Sulfolobus*, for example, Alba2 is a homologue known to preferentially form heterodimers with Alba1 [15]. Comparative analysis of in vitro reconstituted chromatin using Alba1 homodimer and Alba1/2 heterodimer corroborated previous studies that showed Alba heterodimers from *S. solfataricus* form more compact chromatin structures than Alba homodimers [29]. It is known that Alba2 prevents cooperative side-by-side binding of the Alba dimers, which leads to greater DNA compaction [29]. This effect may translate to long-range interactions mediated by Alba heterodimers providing a mechanism to form and fortify tightly organized archaeal chromatin. A comparable binding pattern between Alba heterodimers was also observed in *Aeropyrum pernix* [28], further supporting its potential function in promoting chromatin compaction.

Alba homologues can also be found in eukaryotes [41], although in eukaryotes it is generally involved in non-chromatin functions, such as RNA metabolism and transcriptional regulation [43]. A previous study has also shown that, even in archaea, Alba exhibits RNA-binding activity in vivo [44]. With the knowledge that Alba’s DNA-binding domain exhibits structural similarity with RNase P [45], and that a homologue of the Alba chromatin fold has been observed in bacterial proteins [46] in another context, it can be inferred that Alba was specifically recruited to serve a function in archaeal chromatin organization. In this regard, it will be interesting to know how the role of Alba has affected its other functions within the cell. Currently, it is unknown how the roles of Alba in chromatin organization and metabolic processes is distinguished, though perhaps localization within the cell is a crucial factor in determining its role.

In euryarcheotes, it was observed that Alba is only a minor component of the chromatin, expressed in much lower amounts, compared to histones [45]. Thus, it will be interesting to discover how Alba has acquired a seemingly primary role in chromatin compaction in non-histone-containing archaea such as *S. solfataricus*. Moreover, the extent of the contribution that Alba has on chromatin packaging has yet to be determined in vivo, as experiments have been limited to in vitro assays.

### 4.3. Chromatin Dynamics in S. solfataricus

With the knowledge that Alba may potentially be a primary element in chromatin compaction, this study then explored how other architectural proteins affect Alba-mediated chromatin structures. A previous study by Maruyama et al. [27] observed that histones and Alba from *Thermococcus kodakarensis* compete with each other in DNA binding, suggesting a potential mechanism for regulating archaeal chromatin compaction. Similarly, results from the reconstitution experiments showed that the presence of Cren7 limited the formation of compact higher-order structures with Alba protein. Thus, in addition to its role in inducing local DNA compaction, Cren7 may serve an analogous role in regulating chromatin dynamics in archaea without histones through binding competition, although the exact details of the interplay between Cren7 and Alba remain to be determined.

In bacteria, differential expression of NAPs is known to regulate chromatin dynamics [47]. For instance, in *E. coli*, the expression of the Dps protein is critical in mediating genomic compaction during periods of starvation [21]. In eukaryotes, it is widely known that post-translational modifications such as methylation and acetylation of histones modulate the degree of chromatin compaction [48]. In archaea, it is recognized that the effects of architectural proteins on DNA structure is concentration-dependent, providing a viable mechanism to control chromatin compaction [15,27,28]. However, a previous proteomics study has shown that the expression of different NAPs, at least in *Sulfolobus*, remains stable throughout the growth phases [49]. Previous studies have also reported that post-translational modifications (PTMs), such as acetylation, lower DNA binding affinity of Alba and allow transcription to proceed in vitro [17,32], although subsequent in vivo data have shown that Alba is methylated at the lysine residues, rather than acetylated [50]. While methylation at the lysine residue was shown to have negligible effects on growth and transcription [50], this does not discount the possibility that PTMs are involved in chromatin compaction regulation. Results of this study revealed differential expression of proteins facilitating PTM such as Sir2 across different growth phases; however, further studies are needed to elucidate how PTMs of NAPs are actually regulated in the cell. Although PTMs of NAPs such as Fis can also be observed in bacteria, the effect on chromatin dynamics is not very pronounced [51].

**Figure 9:**
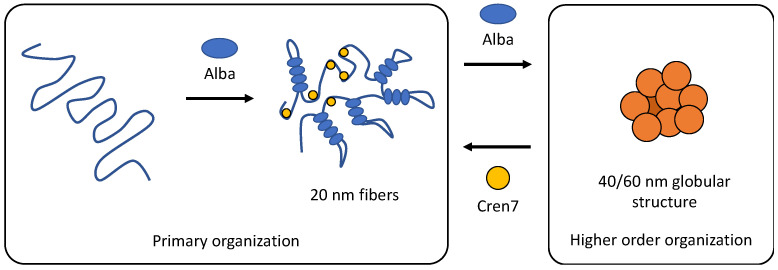
A working model of chromatin compaction driven by Alba, Cren7, and post-translational modifications. Alba serves as the primary driver of compaction, mediating the formation of compact structures on its own. Cren7, on the other hand, competes against Alba in DNA binding, thereby promoting the formation of a looser structure.

Overall, a working model of chromatin compaction and regulation may be derived from this study. The interplay between Alba and Cren7, as well as post-translational modifications that happen to at least one of them, are central to this model (Figure 9). Alba serves as the primary driver of chromatin compaction, whose effect depends on the amount of protein bound to the DNA duplex. Meanwhile, the presence of Cren7 leads to the formation of more open chromatin, making it accessible to processes such as transcription.

In its current form, the proposed model is limited, as it is confined to a minimum set of architectural proteins, and many more proteins are involved in the formation of higher-order structures [42]. Moreover, interactions of other non-architectural DNA-binding proteins such as transcription factors [27,52] should be considered, as they can potentially interact not just with DNA but also with the architectural proteins. Nonetheless, this model serves as a starting point in explaining the mechanistic details of genome organization and chromatin dynamics in archaea.

## Figures and Tables

**Table 1:** List of qPCR primer sequences.

Target Gene	Forward Sequence (5′ → 3′)	Reverse Sequence (3′ → 5′)
*glyA*	AGGACGCAGACTTTGAACCT	AAACTCTAAACACAGCCGCA
*albA*	ACTCTCGATTGCCTTCCGTC	GGAAGAGCTATTAGCAAAGCCG
*albA2*	CAGGTAGGGAAATTTCTAAAGCAG	CGCCTTCTATCTCTAACTTCACTT
*creN7*	ACATGGCAGCTAGTTTCACC	TCTCTACCGAGGAACCTCCC
*Sir2*	AGGTGTGATTGTGGTGGGAT	CAACGGGGTCTCCTCCATAT
*Pat*	ACAGAACACTAGGGATAGGGAC	GCGGAGAAAGTTGCTAGGTT

**Table 2:** Contour length and end-to-end distance measurements of Alba1 and Cren7 nucleoprotein complexes.

Setup	Number of Measured Objects	Contour Length		End-to-End Distance	
		Mean (µm)	SD	Mean (µm)	SD
Naked DNA	25	1.03	0.14	0.364	0.15
6:1 Alba1-DNA	25	0.954	0.20	0.156	0.12
24:1 Alba1-DNA	25	1.20	0.26	0.654	0.20
0.3:1 Cren7-DNA	20	0.948	0.11	0.464	0.20
0.6:1 Cren7-DNA	20	0.899	0.11	0.297	0.15
1:1 Cren7-DNA	20	0.704	0.12	0.373	0.12

## Data Availability

Data is contained within the article.
